# Triplet chemotherapy with docetaxel, cisplatin and S-1 for unresectable advanced squamous cell carcinoma of the esophagus: phase I/II trial results

**DOI:** 10.18632/oncotarget.26614

**Published:** 2019-01-25

**Authors:** Toshiyasu Ojima, Masaki Nakamura, Mikihito Nakamori, Masahiro Katsuda, Keiji Hayata, Junya Kitadani, Shimpei Maruoka, Toshio Shimokawa, Hiroki Yamaue

**Affiliations:** ^1^ Second Department of Surgery, School of Medicine, Wakayama Medical University, Wakayama, Japan; ^2^ Clinical Study Support Center, Wakayama Medical University, Wakayama, Japan

**Keywords:** squamous cell carcinoma, esophageal cancer, docetaxel, cisplatin, S-1

## Abstract

**Background:**

Although triplet regimen of docetaxel, cisplatin, and 5-FU (DCF) reportedly yields high response rates for metastatic squamous cell carcinoma of the esophagus (SCCE), it has severe toxicity. In our previous phase II trial, grade 3/4 toxicities of neutropenia occurred in 68.8% of the patients. Development of chemotherapeutic regimen that does not impair quality of life of the patients with metastatic SCCE is therefore needed. A novel chemotherapeutic regimen combining docetaxel, cisplatin, and alternate-day administration of S-1 (modified DCS) may be associated with reduction of severe adverse effects.

**Methods:**

This study is a single center phase I/II trial of chemotherapy using modified DCS regimen for patients with recurrent/unresectable SCCE. The phase I trial adopts a ‘3 + 3 patient cohort’, dose-escalating study design. In the phase II trial, the primary endpoint is evaluation of the overall response rate (ORR). Secondary endpoints are evaluation of drug-related toxicity, overall survival (OS), and progression-free survival (PFS).

**Results:**

In the phase I trial, the recommended dose for docetaxel, cisplatin, and S-1 were 40 mg/m^2^ (day 1), 50 mg/m^2^ (day 1), and 80 mg/m^2^/day, respectively. In the phase II trial (n = 50), the ORR was 54 %. The median OS and PFS were 10 and 4 months, respectively. Grade 3/4 adverse events included neutropenia (26%), leukopenia (14%), anorexia (10%) and febrile neutropenia (6%).

**Conclusion:**

The modified DCS therapy for patients with advanced SCCE is feasible and safe in both chemotherapeutic and perioperative periods.

Registration number: UMIN000016364.

## INTRODUCTION

Squamous cell carcinoma of the esophagus (SCCE) is common in Asia, and is a malignant tumor with poor prognosis. The primary treatment for patients with resectable SCCE is radical surgery. At diagnosis, however, more than half of patients are unsuitable candidates for radical surgery because of the high frequency of unresectable primary disease or distant metastases [1, 2]. In these medically unfit patients, definitive chemotherapy has been established as a standard treatment. The most widely used chemotherapeutic regimen for metastatic SCCE is the combination of cisplatin (CDDP) plus 5-FU (CF) [3, 4], however, the effective response is only 25-35% [3–5].

In recent years, triplet regimen that adds docetaxel to CF (DCF) has been reported to yield high response rates for metastatic SCCE [6–11]. Our previous phase II trial of 48 patients with metastatic SCCE had DCF regimen response rate of 62.5% [12]. However, DCF regimen has severe toxicity [6–11]. In our phase II trial, grade 3 or higher toxicities of neutropenia and febrile neutropenia occurred in 68.8% and 14.6% of the patients, respectively [12]. All patients who underwent DCF regimen required long-term hospitalization. Development of a new chemotherapeutic regimen that does not negatively affect quality of life (QOL) of patients with metastatic SCCE is urgently needed.

S-1 is an oral fluoropyrimidine derivative consisting of tegafur, gimeracil, and oteracil. It has been suggested as a key drug for chemotherapy of advanced gastric cancer [13]. S-1 has also been approved as a single or combination therapy for SCCE, and has reported good results [14–16]. An S-1 treatment regimen used in gastric cancer patients was four weeks of twice-daily administration followed by two weeks of no treatment [13]. In the SPIRITS trial using this dosage regimen of S-1 alone, the frequency of grade 3/4 toxicities was 25% in patients [13].

To reduce the incidence of toxicity, the efficacy and safety of an alternate-day administration of S-1 were investigated in patients with gastric cancer, with lower reported adverse effects [17, 18]. Our randomized phase II study for metastatic pancreatic cancer showed that the incidence of grade 3/4 hematological toxicities was 4.2% in patients treated with alternate-day administration of S-1 [19]. We therefore hypothesized that an alternate-day administration with S-1 as part of triplet regimen for metastatic SCCE may be associated with reduction of severe adverse effects. No prospective studies have yet investigated the effectiveness of combining docetaxel, CDDP, and administration of S-1 on alternate days (modified DCS) for advanced SCCE.

This novel prospective phase I/II trial examines the efficacy and toxicity of modified DCS for patients with advanced SCCE.

## RESULTS

### Phase I

Between January and March 2015, nine patients were enrolled in the phase I trial. All enrolled patients were males, their median age was 64 years (50 - 80). The toxicities are summarized in Table 1. At level 3 (dose of docetaxel 50 mg/m^2^), two patients had grade 4 neutropenia, one patient had grade 4 leukopenia, and one patient had febrile neutropenia. Dose level 3 was defined as the maximum tolerated dose (MTD), and dose level 2 (the dose of docetaxel was 40 mg/mm^2^) was adopted as the recommended dose (RD).

**Table 1:** Hematological and non-hematological toxicities in phase I study

Categories	Level 1 (n = 3)			Level 2 (n = 3)			Level 3 (n = 3)		
	1-2^*^	3^*^	4^*^	1-2	3	4	1-2	3	4
Leukopenia	0	0	0	2	0	0	1	0	1^**^
Neutropenia	0	0	0	1	0	0	0	0	2^**^
Anemia	0	0	0	2	0	0	0	0	0
Thrombocytopenia	0	0	0	0	0	0	0	0	0
Anorexia	2	0	0	0	1	0	0	2	0
Diarrhea	0	0	0	1	0	0	0	0	0
Stomatitis	0	0	0	1	0	0	0	0	0
Alopecia	1	0	0	1	0	0	1	0	0
Febrile neutropenia	0	0	0	0	0	0	0	1^**^	0

^*^ NCI-CTC: National Cancer Institute Common Toxicity Criteria

^**^ Dose-limiting toxicity

### Phase II

#### Patients characteristics

Between April 2015 and November 2017, 50 patients were enrolled in the phase II trial. Table 2 summarizes the detailed characteristics of these 44 male and six female patients, who had a median age of 68.5 years. Of the 50 patients, 34 were new cases and 16 patients had recurrent tumors. All patients had metastatic disease; 36 had metastasis to a lymph node, ten had liver metastasis, six had lung metastasis, four had bone metastasis, two had peritoneal dissemination, and one had adrenal metastasis. Eighteen patients had received one or more previous treatments: one patient received chemoradiotherapy (radiation with CF therapy), seventeen patients received chemotherapy, fourteen patients received neo adjuvant chemotherapy (DCF therapy) with planned esophagectomy, and three patients received the definitive DCF therapy for metastatic diseases. In this phase II trial, no patient had salvage or conversion surgery after modified DCS therapy.

**Table 2:** Phase II patient characteristics (n = 50)

Characteristics	No. of patients
Gender, male/female	44/6
Age in years, median (range)	68.5 (42-80)
Performance status (ECOG2) 0/1/2	38/12/0
Disease status, recurrent/unresectable	16/34
Previous treatment, yes/no	18/32
Previous treatment, Chemoradiotherapy/other chemotherapy	1/17
Site of metastasis (overlapping), Lymph nodes/liver/lung/bone/peritoneum/adrenal	36/10/6/4/2/1

Abbreviations: ECOG: Eastern Cooperative Oncology Group.

#### Responses

The median number of cycles delivered per patient was four cycles (range: 1-13). All 50 patients were included in the response analyses (Table 3). A clinical complete response (CR) was seen in five patients (10.0%) and a partial response (PR) was seen in 22 patients (44.0%) for an overall response rate (ORR) of 54.0%. Eleven patients (22.0%) had stable disease (SD), and twelve patients (24.0%) had progressive disease (PD).

**Table 3:** The overall response rates (n = 50)

Responses	No. of patients (%)
Complete response (CR)	5 (10.0)
Partial response (PR)	22 (44.0)
Stable disease (SD)	11 (22.0)
Progressive disease (PD)	12 (24.0)
Effective response (CR + PR)	27 (54.0)

#### Survival

**Figure 1:**
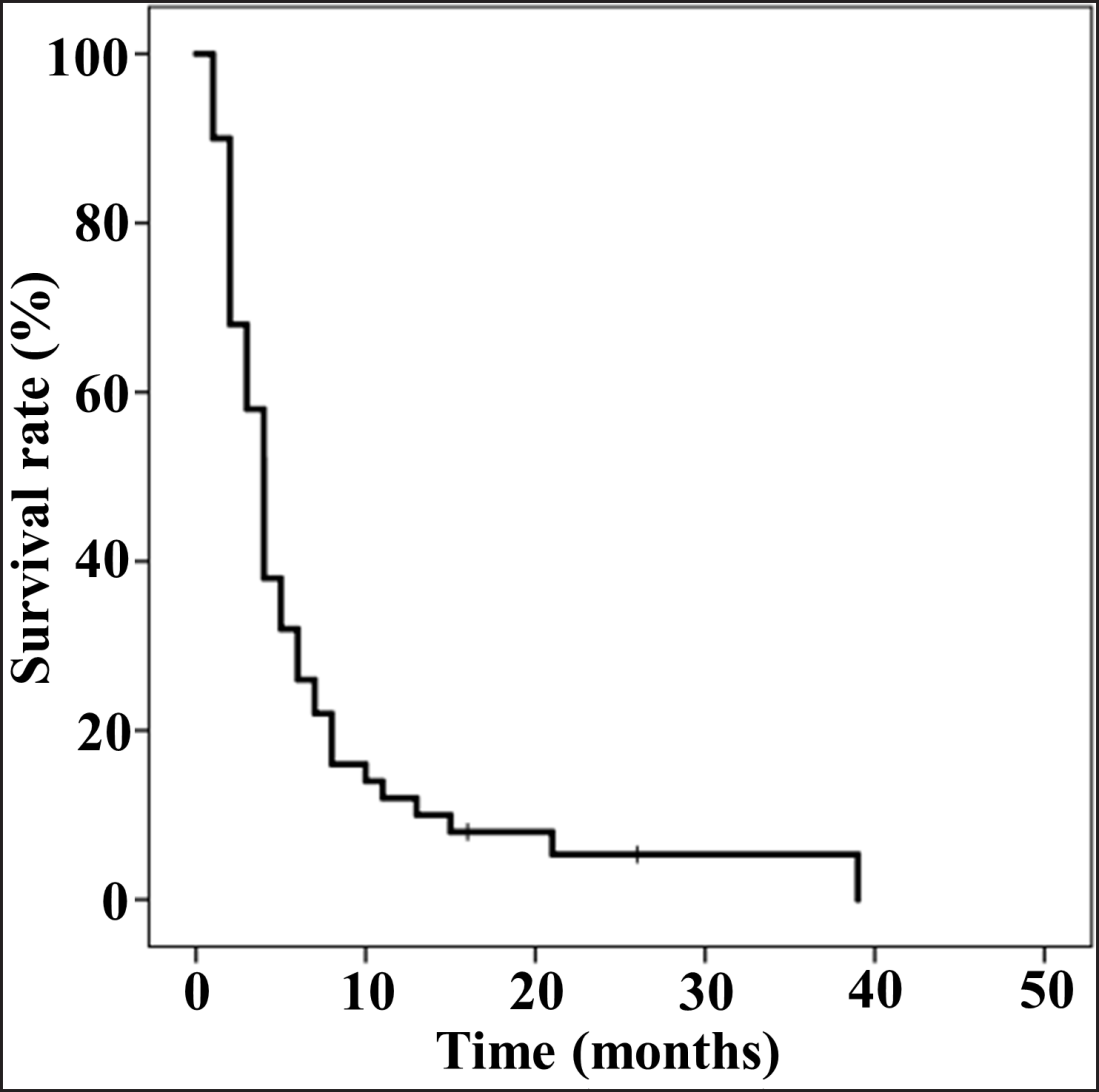

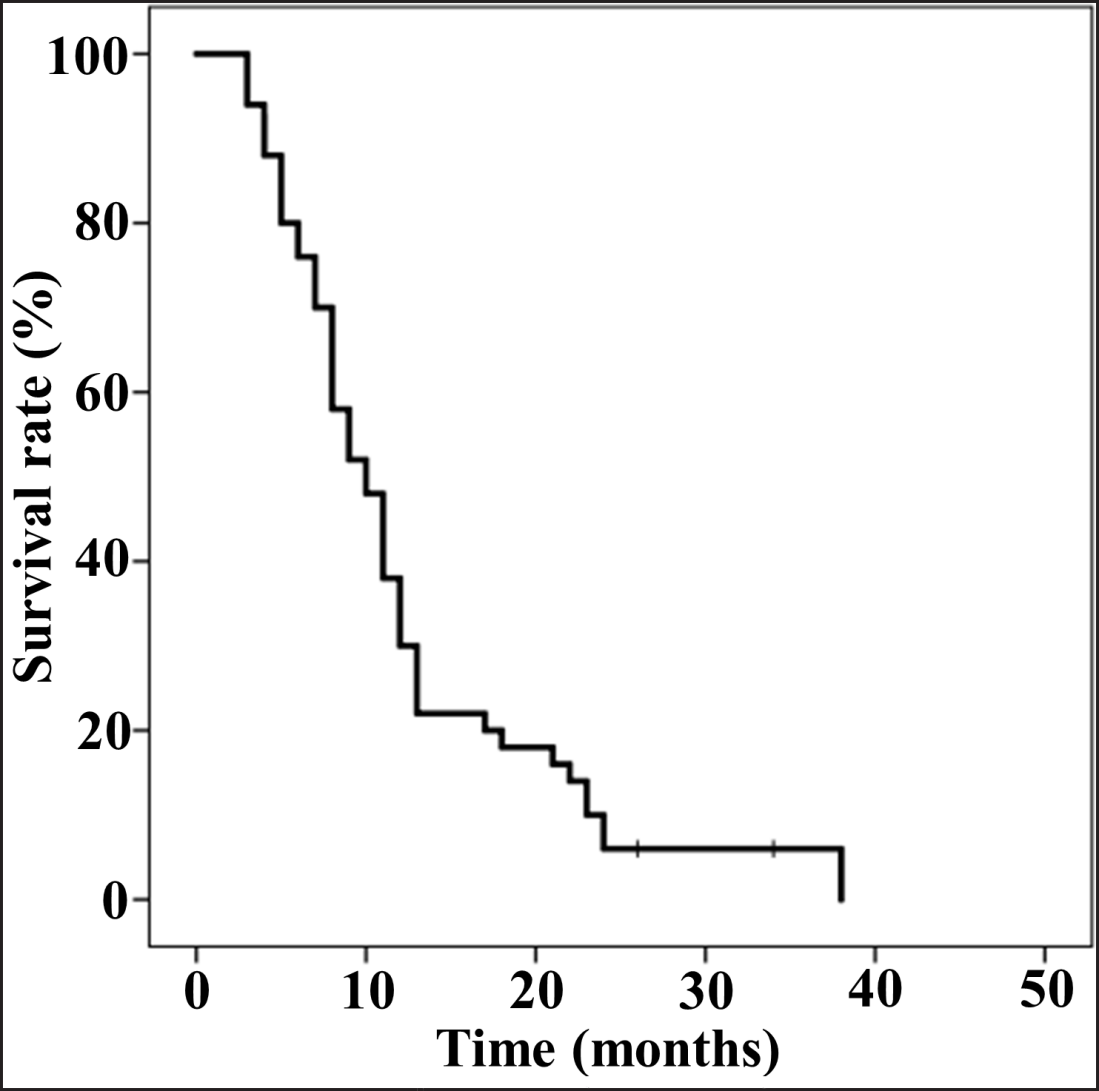
The progression-free survival (PFS) and overall survival (OS) of 50 patients **(A)** PFS was analyzed by the Kaplan-Meier method. Median PFS was 4 months (95% confidence interval [CI], 3.3-4.7). **(B)** OS was analyzed by the Kaplan-Meier method. Median OS was 10 months (95% CI, 7.9-12.1).

Survival analysis was conducted on all 50 patients. As shown in Figure 1A, these patients exhibited a median progression-free survival (PFS) of four months (95% confidence interval [CI], 3.3 to 4.7 months). The median overall survival (OS) was 10 months (95% CI, 7.9 to 12.1 months) (Figure 1B). The one-year PFS and OS rates were 12.0% and 30.0%, and the 2-year PFS and OS rates were 5.3% and 6.0%, respectively (Figure 1AB).

#### Toxicity

The toxicity observed in the phase II trial is shown in Table 4. There were no treatment-related deaths in this trial. Grade 3/4 leukopenia and neutropenia were observed in 14.0% and 26.0% of the patients, respectively. All patients improved relatively quickly by administration of granulocyte-colony stimulating factor (G-CSF). As representative non-hematological toxicities, febrile neutropenia and grade 3 anorexia were detected in 6.0% and 10.0% of the patients, respectively. No patients had grade 3/4 nausea.

**Table 4:** The hematological and non-hematological toxicities in phase II study (n = 50)

Categories		NCI-CTC Grade			Grade 3/4 (%)
	1	2	3	4	
Leukopenia	2	4	6	1	14.0
Neutropenia	4	2	9	4	26.0
Anemia	1	3	0	0	0
Thrombocytopenia	1	2	1	0	2.0
Anorexia	1	5	5	0	10.0
Diarrhea	2	2	1	0	2.0
Nausea	2	2	0	0	0
Stomatitis	3	1	0	0	0
Alopecia	1	3	0	0	0
Febrile neutropenia	0	0	3	0	6.0

Abbreviations: NCI-CTC, National Cancer Institute Common Toxicity Criteria.

The association between treatment toxicity and clinical antitumor effect was not found in this trial.

## DISCUSSION

This phase I/II trial was carried out to investigate the efficacy and safety of modified DCS regimen for advanced SCCE, including both unresectable and recurring cases. This study was the first clinical trial using the combination of docetaxel, CDDP, and S-1 for advanced SCCE. The novelty of our treatment regimen was the alternate-day administration of S-1.

We found higher antitumor activity compared with the results of previous studies using the CF regimen [3–5]. The ORR of 54% in our trial was comparable with that reported previously using DCF regimens in Japan (44.8% and 66.6%, respectively), in Australia (47%), and in USA (34%) [6, 7, 10, 20]. The median OS of 10 months was also similar to those of the DCF regimen in Japan (9, 10.6, and 13 months, respectively), in Australia (11.2 months), and in USA (8.9 months) [6, 7, 9, 10, 20]. Our previous phase II trial of 48 patients with metastatic SCCE showed that the response rate of DCF regimen was 62.5% and the median OS was 13 months [12]. Considering these previous data, our modified DCS regimen maintained its efficacy even with the dose reduction.

We compared the clinical response and survival between the patients with and without previous DCF chemotherapy. The ORR was 57.6% in patients without previous DCF chemotherapy, and 47.1% in patients with previous DCF chemotherapy. The ORR did not differ significantly between the two groups (data not shown). The median OS was 10 months among patients without previous DCF chemotherapy, and 11 months among patients with previous DCF chemotherapy (data not shown). Interestingly, our results showed that this modified DCS regimen was well tolerated regardless of whether patients had received previous DCF chemotherapy. Therefore, we consider that this modified DCS therapy is useful not only as a first-line treatment, but also as a second or third-line treatment regimen.

In patients with advanced SCCE, best supportive care can be an option for treatment. In our institute, however, the median OS of the best supportive care patients was only 4 months. Therefore, we consider that salvage chemotherapy is needed even in the case with aggressive advanced SCCE.

Safe and long-term chemotherapy without impairing QOL is important for improving the clinical outcomes of SCCE. In previous studies with DCF regimen, hematological toxicities were the most frequent and important adverse events [6–10, 20]. Indeed, our previous phase II trial using DCF regimen showed that 64.6%, 68.8%, and 14.6% of patients had grade 3/4 leukopenia, neutropenia, and febrile neutropenia, respectively [12]. In this modified DCS regimen, hematologic toxicity was significantly less than in DCF regimen, and moreover, the incidence rate of non-hematological toxicity was very low. Although all the patients who underwent DCF regimen required long-term hospitalization, the majority of patients in this trial were manageable by just three days of hospitalization per cycle. The main factor of reduction of adverse events was alternate-day administration of S-1 instead of continuous administration of 5-FU for five days. Alternate-day administration of S-1 was developed with the rationale that it attenuates gastrointestinal toxicity and bone marrow suppression without decreasing the cancer cell killing effect [21]. Modified chemotherapy including alternate-day administration of S-1 was used not only in patients with metastatic SCCE but also in patients with gastric cancer, colorectal cancer and pancreatic cancer, and many papers have proved to reduce the toxicity [17–19, 22]. This modified DCS regimen may be especially useful in chemotherapy for frail and elderly patients with advanced SCCE. According to our toxicity results, preservation of QOL and clinical benefit favored modified DCS over DCF. A prospective randomized controlled trial (RCT) to evaluate the toxicity and oncological outcomes of patients with unresectable SCCE treated with DCF or modified DCS is required.

This study had several limitations. It was a phase I/II study without RCT and it was conducted at a single institution. This study also included a small sample size. Findings from this trial do not allow established clinical application, but rather serve to inform the need for larger multicenter phase III RCT of modified DCS regimen for patients with advanced SCCE.

In conclusion, the present trial suggests that modified DCS therapy for patients with advanced SCCE is feasible and safe in both chemotherapeutic and perioperative periods.

## PATIENTS AND METHODS

A protocol paper for this trial was previously published [23].

### Study design

This study was designed as an open-label, single center phase I/II trial of chemotherapy using modified DCS regimen for patients with recurrent/unresectable SCCE. We used a two-stage design. Phase I was undertaken to determine the MTD and RD. In the phase II trial, the primary endpoint was to evaluate the overall response rate (Response Evaluation Criteria in Solid Tumors [RECIST] 1.1 [24]). Secondary endpoints were to evaluate drug-related toxicity (National Cancer Institute - Common Toxicity Criteria [NCI-CTC] 4.0 [25]), OS, and PFS. The study was carried out in accordance with the Declaration of Helsinki. This study was approved by the ethical committee on human research at Wakayama Medical University Hospital (WMUH), and was registered on the University Hospital Medical Information Network Clinical Trials Registry (UMIN000016364). All patients provided written informed consent.

### Patient eligibility criteria

Patients were eligible if they had histologically confirmed SCCE; had locally advanced (T4) and/or metastatic (M1) esophageal cancer [26]; had recurrent esophageal cancer; were aged between 20-85 years; and had Eastern Cooperative Oncology Group (ECOG) performance status (PS) of 0-1. Furthermore, to fulfill eligibility criteria, patients were required to have an absolute 2,000/mm^3^ < White blood cell count < 12,000/mm^3^, neutrophil count > 1,500/mm^3^, hemoglobin > 8.0 g/dl, platelet count > 100,000/mm^3^, total bilirubin < 1.5 mg/dL, aspartate aminotransferase and alanine aminotransferase < 150 IU/L, and creatinine < 1.5 mg/dL. Patients with any of the following conditions were excluded: active or uncontrolled infection, myocardial infarction within the previous three months, uncontrolled diabetes mellitus or hypertension, or clinically apparent central nervous system metastases.

### Treatment

**Figure 2:**
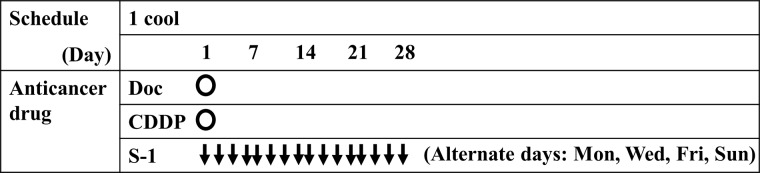
Treatment design of chemotherapy with docetaxel, cisplatin and S-1 Doc: Docetaxel, CDDP: cisplatin.

The phase I trial adopted a ‘3 + 3’ patient cohort, dose-escalating study design. The dose of docetaxel was escalated as follows: level 1: 30 mg/m^2^, level 2: 40 mg/m^2^, and level 3: 50 mg/m^2^. This was intravenously infused over 2 hr on day 1 of the trial. CDDP was administered at a fixed dose of 50 mg/m^2^ infused over 4 hr on day 1. S-1 was administered at a fixed dose of 80 mg/m^2^/day on alternate days (twice daily on Monday, Wednesday, Friday and Sunday between days 1 and 28 of a 28-day cycle) [17, 18]. This regimen was composed of one course repeated every four weeks (Figure 2). Dose-limiting toxicity (DLT) was defined as either grade 4 leukopenia or neutropenia; grade 3 or greater neutropenia with fever; grade 4 thrombocytopenia, or non-hematological toxicity of grade 3 except for anorexia, nausea, vomiting and alopecia. A group of three patients were each given the same dose level, and if no DLT was observed in any of them, the dose was increased to the next level. If DLT was observed in one of the three patients at a particular level, three additional patients were treated at the same dose level. If DLT was observed in at least three of the total six patients, the dose was judged to be the MTD. Also, if DLT was observed in two of three patients at any level, this dose level was judged to be MTD. The dose one level below the MTD was finally selected as the RD. The phase II trial used the RD determined in the phase I trial. The treatment regimen was repeated every four weeks. The dose of docetaxel was modified according to the degree of myelosuppression. Dose modification was based on the worst toxicity observed during the previous course. For grade 4 neutropenia or febrile neutropenia, the docetaxel dose was reduced by one level (reduction of 10 mg/m^2^) in the next cycle after recovery to grade 1. This treatment regimen was repeated without a limit, unless progression, unacceptable level of toxicity, or patient refusal occur. When patients were withdrawn from the trial, the subsequent treatment was not defined.

### Assessment

The radiologic tumor response was evaluated by the RECIST 1.1 using enhanced computed tomography (CT) scans every four weeks. For safety assessment, adverse events were scored using the NCI-CTC 4.0. Complete blood cell counts were taken at least once per week. Biochemical panels with renal and liver function tests were monitored before each cycle of chemotherapy. PFS was calculated as the time of the first administration of chemotherapy to the first confirmed disease progression or until death from any cause. OS was defined as the time from the first administration of chemotherapy to the date of death from any cause.

### Statistical analysis

In the phase II trial, a total of 50 samples (45, plus 5 dropout cases) were required. The sample size was calculated to confirm the null hypothesis that the 95% confidence interval of the expected ORR (55%) would be less than 30% under conditions of α error of 0.05 and β error of 0.2. These estimates were calculated by a reliable clinical statistician (Prof. T. Shimokawa). Quantitative results were expressed as medians and ranges. Survival curves were computed by Kaplan-Meier method. SPSS version 24.0 software program (SPSS Inc., Chicago, USA) was used for all statistical analyses. The Clinical Study Support Center at WMUH was responsible for data management, central monitoring, and statistical analysis.

## Abbreviations

SCCE: squamous cell carcinoma of the esophagus
CDDP: cisplatin
CF: cisplatin plus 5-FU
DCF: docetaxel, cisplatin, and 5-FU
QOL: quality of life
DCS: docetaxel, cisplatin, and S-1
MTD: maximum tolerated dose
RD: recommended dose
CR: complete response
PR: partial response
ORR: overall response rate
SD: stable disease
PD: progressive disease
PFS: progression-free survival
CI: confidence interval
OS: overall survival
G-CSF: granulocyte-colony stimulating factor
RCT: randomized controlled trial
RECIST: Response Evaluation Criteria in Solid Tumors
NCI-CTC: National Cancer Institute - Common Toxicity Criteria
WMUH: Wakayama Medical University Hospital
ECOG: Eastern Cooperative Oncology Group
PS: performance status
DLT: dose-limiting toxicity

## References

[1] Sagar PM, Gauperaa T, Sue-Ling H, McMahon MJ, Johnston D (1994). An audit of the treatment of cancer of the oesophagus. Gut.

[2] Pennathur A, Gibson MK, Jobe BA, Luketich JD (2013). Oesophageal carcinoma. Lancet.

[3] Hayashi K, Ando N, Watanabe H, Ide H, Nagai K, Aoyama N, Takiyama W, Ishida K, Isono K, Makuuchi H, Imamura M, Shinoda M, Ikeuchi S (2001). Phase II evaluation of protracted infusion of cisplatin and 5-fluorouracil in advanced squamous cell carcinoma of the esophagus: a Japan Esophageal Oncology Group (JEOG) Trial (JCOG9407). Jpn J Clin Oncol.

[4] Iizuka T, Kakegawa T, Ide H, Ando N, Watanabe H, Tanaka O, Takagi I, Isono K, Ishida K, Arimori M, Endo M, Fukushima M (1992). Phase II evaluation of cisplatin and 5-fluorouracil in advanced squamous cell carcinoma of the esophagus: a Japanese Esophageal Oncology Group Trial. Jpn J Clin Oncol.

[5] Bleiberg H, Conroy T, Paillot B, Lacave AJ, Blijham G, Jacob JH, Bedenne L, Namer M, De Besi P, Gay F, Collette L, Sahmoud T (1997). Randomised phase II study of cisplatin and 5-fluorouracil (5-FU) versus cisplatin alone in advanced squamous cell oesophageal cancer. Eur J Cancer.

[6] Takahashi H, Arimura Y, Yamashita K, Okahara S, Tanuma T, Kodaira J, Hokari K, Tsukagoshi H, Shinomura Y, Hosokawa M (2010). Phase I/II study of docetaxel/cisplatin/fluorouracil combination chemotherapy against metastatic esophageal squamous cell carcinoma. J Thorac Oncol.

[7] Tebbutt NC, Cummins MM, Sourjina T, Strickland A, Van Hazel G, Ganju V, Gibbs D, Stockler M, Gebski V, Zalcberg J, Australasian Gastro-Intestinal Trials Group (2010). Randomised, non-comparative phase II study of weekly docetaxel with cisplatin and 5-fluorouracil or with capecitabine in oesophagogastric cancer: the AGITG ATTAX trial. Br J Cancer.

[8] Yamasaki M, Miyata H, Tanaka K, Shiraishi O, Motoori M, Peng YF, Yasuda T, Yano M, Shiozaki H, Mori M, Doki Y (2011). Multicenter phase I/II study of docetaxel, cisplatin and fluorouracil combination chemotherapy in patients with advanced or recurrent squamous cell carcinoma of the esophagus. Oncology.

[9] Osaka Y, Shinohara M, Hoshino S, Ogata T, Takagi Y, Tsuchida A, Aoki T (2011). Phase II study of combined chemotherapy with docetaxel, CDDP and 5-FU for highly advanced esophageal cancer. Anticancer Res.

[10] Tamura S, Imano M, Takiuchi H, Kobayashi K, Imamoto H, Miki H, Goto Y, Aoki T, Peng YF, Tsujinaka T, Furukawa H, Osaka Gastrointestinal Cancer Chemotherapy Study Group (2012). Phase II study of docetaxel, cisplatin and 5-fluorouracil (DCF) for metastatic esophageal cancer (OGSG 0403). Anticancer Res.

[11] Hironaka S, Tsubosa Y, Mizusawa J, Kii T, Kato K, Tsushima T, Chin K, Tomori A, Okuno T, Taniki T, Ura T, Matsushita H, Kojima T, Japan Esophageal Oncology Group/Japan Clinical Oncology Group (2014). Phase I/II trial of 2-weekly docetaxel combined with cisplatin plus fluorouracil in metastatic esophageal cancer (JCOG0807). Cancer Sci.

[12] Ojima T, Nakamori M, Nakamura M, Katsuda M, Hayata K, Matsumura S, Iwahashi M, Yamaue H (2017). Phase I/II study of divided-dose docetaxel, cisplatin and fluorouracil for patients with recurrent or metastatic squamous cell carcinoma of the esophagus. Dis Esophagus.

[13] Koizumi W, Narahara H, Hara T, Takagane A, Akiya T, Takagi M, Miyashita K, Nishizaki T, Kobayashi O, Takiyama W, Toh Y, Nagaie T, Takagi S (2008). S-1 plus cisplatin versus S-1 alone for first-line treatment of advanced gastric cancer (SPIRITStrial): a phase III trial. Lancet Oncol.

[14] Tahara M, Fuse N, Mizusawa J, Sato A, Nihei K, Kanato K, Kato K, Yamazaki K, Muro K, Takaishi H, Boku N, Ohtsu A (2015). Phase I/II trial of chemoradiotherapy with concurrent S-1 and cisplatin for clinical stage II/III esophageal carcinoma (JCOG 0604). Cancer Sci.

[15] Chang H, Shin SK, Cho BC, Lee CG, Kim CB, Kim DJ, Lee JG, Hur J, Lee CY, Bae MK, Kim HR, Lee SK, Park JC (2014). A prospective phase II trial of S-1 and cisplatin-based chemoradiotherapy for locoregionally advanced esophageal cancer. Cancer Chemother Pharmacol.

[16] Iwase H, Shimada M, Tsuzuki T, Hirashima N, Okeya M, Hibino Y, Ryuge N, Yokoi M, Kida Y, Kuno T, Tanaka Y, Kato B, Esaki M (2013). Concurrent chemoradiotherapy with a novel fluoropyrimidine, S-1, and cisplatin for locally advanced esophageal cancer: long-term results of a phase II trial. Oncology.

[17] Sakuma K, Hosoya Y, Arai W, Haruta H, Ui T, Kurashina K, Saito S, Hirashima Y, Yokoyama T, Zuiki T, Hyodo M, Nagai H, Yasuda Y, Shirasaka T (2010). Alternate-day treatment with S-1 in patients with gastric cancer: a retrospective study of strategies for reducing toxicity. Int J Clin Oncol.

[18] Arai W, Hosoya Y, Hyodo M, Yokoyama T, Hirashima Y, Yasuda Y, Nagai H, Shirasaka T (2004). Alternate-day oral therapy with TS-1 for advanced gastric cancer. Int J Clin Oncol.

[19] Yamaue H, Shimizu A, Hagiwara Y, Sho M, Yanagimoto H, Nakamori S, Ueno H, Ishii H, Kitano M, Sugimori K, Maguchi H, Ohkawa S, Imaoka H (2017). Multicenter, randomized, open-label Phase II study comparing S-1 alternate-day oral therapy with the standard daily regimen as a first-line treatment in patients with unresectable advanced pancreatic cancer. Cancer Chemother Pharmacol.

[20] Overman MJ, Kazmi SM, Jhamb J, Lin E, Yao JC, Abbruzzese JL, Ho L, Ajani J, Phan A (2010). Weekly docetaxel, cisplatin, and 5-fluorouracil as initial therapy for patients with advanced gastric and esophageal cancer. Cancer.

[21] Shirasaka T, Shimamoto Y, Fukushima M (1993). Inhibition by oxonic acid of gastrointestinal toxicity of 5-fluorouracil without loss of its antitumor activity in rats. Cancer Res.

[22] Matsuda C, Honda M, Tanaka C, Kondo K, Takahashi T, Kosugi C, Tokunaga Y, Takemoto H, Kim HM, Sakamoto J, Oba K, Mishima H (2018). A phase II study of bevacizumab and irinotecan plus alternate-day S-1 as a second-line therapy in patients with metastatic colorectal cancer: the AIRS study. Cancer Chemother Pharmacol.

[23] Ojima T, Nakamura M, Nakamori M, Katsuda M, Hayata K, Maruoka S, Shimokawa T, Yamaue H (2018). Phase I/II Trial of Chemotherapy with Docetaxel, Cisplatin, and S-1 for Unresectable Advanced Squamous Cell Carcinoma of the Esophagus. Oncology.

[24] Eisenhauer EA, Therasse P, Bogaerts J, Schwartz LH, Sargent D, Ford R, Dancey J, Arbuck S, Gwyther S, Mooney M, Rubinstein L, Shankar L, Dodd L (2009). New response evaluation criteria in solid tumours: revised RECIST guideline (version 1.1). Eur J Cancer.

[25] National Cancer Institute USDHHS (2009). Common Terminology Criteria for Adverse Events (CTCAE), Version 4.0.

[26] Brierley JD, Gospodarowicz MK, Wittekind C (2017). TNM Classification of Malignant Tumours.

